# The Phenomenology of Objectification in and Through Medical Practice and Technology Development

**DOI:** 10.1093/jmp/jhad007

**Published:** 2023-04-20

**Authors:** Fredrik Svenaeus

**Affiliations:** Södertörn University, Södertörn, Sweden

**Keywords:** *genetic diagnosis*, *lived body*, *medical hermeneutics*, *objectification*, *phenomenology of medicine*, *quantified self*

## Abstract

Objectification is a real problem in medicine that can lead to bad medical practice or, in the worst case, dehumanization of the patient. Nevertheless, objectification also plays a major and necessary role in medicine: the patient’s body should be viewed as a biological organism in order to find diseases and be able to cure them. Listening to the patient’s illness story should not be replaced, but, indeed, developed by the physical examination of his body searching for the causes of his complaints. Whereas phenomenologists have so far mainly been identifying the back sides of objectification in medicine, in this paper the aim is to analyze differences between detrimental objectifications and objectifications that do not deprive the patient of his subjectivity but, rather, at least in some cases, may lead the patient to feel more at home with his body.

## I. INTRODUCTION

A major issue for phenomenological analyses in the areas of medicine and bioethics has been the danger of objectifying the patient in the medical encounter by viewing and handling him mainly or merely as a physical body to be scientifically investigated (Gadamer, 1996; Svenaeus, 2000; Young, 2005; Aho and Aho, 2008). The patient is not only a bodily specimen of a *disease* to be diagnosed and treated, but first and foremost an *ill person* to be listened to and helped. Phenomenologists of medicine have pointed out and elaborated on this distinction in various ways since at least the 1990s (Carel, 2008; Leder, 2016; Svenaeus, 2017). According to phenomenologists, the increasing number of different forms of medical objectification in and outside the clinic entrenches risks regarding bodily alienation. The body becomes a foreign territory rather than the patient’s home ground by way of such objectifications, a development which was inaugurated already with the birth of modern medicine and what Michel Foucault famously dubbed “the medical gaze” (Foucault, 1994; Slatman, 2014). The making visible of the insides of living human bodies was made possible towards the end of the 19th century with technologies such as endoscopy and X-rays. These were used not only in medical practice but also outside the clinic, generating feelings of surprise, astonishment, and horror among the public (van Dijck, 2005). The distribution and application of various forms of medical technology outside the walls of the hospital has continued to this day, including developments such as the quantified-self movement, which I will return to below (Topol, 2015).

Objectification is no doubt a real problem in medicine and it can lead to bad medical practice or, in the worst case, dehumanization of the patient if not enveloped by a personalistic attitude. Nevertheless, objectification also plays a major and necessary role in medicine, the patient’s body *should* be viewed as a (malfunctioning) biological organism in order to find diseases and be able to cure them. The listening to the patient’s illness story should not be replaced, but, indeed, *developed* by the physical examination of his body searching for the causes of his complaints. Whereas phenomenologists have so far been keen on identifying the negative aspects of objectification in medicine, a more balanced strategy might be to discuss and analyze differences between medical objectifications that make the patient feel like being merely a body and the objectifications that do not deprive the patient of his subjectivity. This is what I intend to do in this paper. “Bad objectification” makes the patient feel like being a body *instead* of being a person, while “good objectification” makes the patient feel like a body but not in a way that is detrimental for his personhood. In some cases, which I will come back to, such good forms of objectification can even make the patient feel more at home with himself by incorporating a richer understanding of what goes on in the body.

## II. MEDICAL PRACTICE AND OBJECTIFICATION

When we visit a health care center or hospital we *expect* to be objectified in a number of ways. Indeed, as I will later discuss, we have in many cases already begun a project of self-objectification assisted by Google searches on our symptoms when we enter the clinic. If I seek help for stomach aches, I do not only expect the doctor to take an interest in my description of the pains and my stressful life situation. I also expect her to conduct a physical examination of my body and collect specimens of blood and/or stools for laboratory tests. Further, I expect her to refer me to a specialist for a CT scan and a gastro- or colonoscopy to detect potential diseases, such as cancer, if she suspects such a cause of my pains. These procedures are not alienating or dehumanizing in themselves, they are only so if the health care staff does not *first and foremost* approach me as a worried and suffering person who they are there to help and take care of. If the doctors and nurses explain to the patient why they are performing the different diagnostic procedures – and do not lose sight of the person and his feelings and thoughts in the situations in which medical technologies are being applied – the focus upon the body as a potentially diseased organism could not only be handled by the patient, the focus is also something that is expected by him. The medical-professional attitude to bodily complaints presented by the patient could, in such situations, not only be necessary to find diseases and apply treatments to cure them, it could also, when adopted by the patient, make him feel relieved and less embarrassed about his bodily state, and also more in control as concerns his future life situation. As already Nietzsche noted in *The Gay Science*, there is something liberating and empowering at work in the process of establishing a distance and order of command between oneself and the malfunctioning body:

*My dog. –* I have given a name to my pain and call it “dog”. It is just as faithful, just as obtrusive and shameless, just as entertaining, just as clever as any other dog – and I can scold it and vent my bad mood on it, as others do with their dogs, (1974, 249).

Among phenomenologists, the patient’s point of view, as opposed to that of the doctor, is taken into account by talking about the “lived body” in contrast to the “physical body.” There is a crucial difference between *existing* as a body and *having* a body – in German between being a “Leib” and a “Körper” – since the body is my way of *being* in the world in contrast to being a physical thing that *belongs* to this world (Leder, 1990; Merleau-Ponty, 2012). The physical body is a biological organism and therefore a *living* body, but it is not *lived*, or, rather, at the point at which we switch the perspective from a scientific outside point of view to the person’s point of view who animates this physical body, we are talking about the lived in contrast to the living body. The lived body consists in a feeling of being alive and present in an environment, but since our attention is normally directed towards things that we perceive and do in the world, this bodily experience recedes into the background. This may change if the lived body suddenly stands out calling for our focused attention, as happens in pain or nausea, but also by way of positive bodily feelings that may occur in activities such as making love, playing sports, or eating and drinking delicious food and wine (Zeiler, 2010). The way the lived body is brought to our attention under such circumstances is not a form of objectification, rather it is a form of intensive *subjectification* by which the bodily aspects of our personal being are brought to light (Svenaeus, 2015). The subjectification in question could nevertheless turn into an alienating experience – when the body hurts and/or resists our efforts to perceive and do things in the world we are currently inhabiting with others – but the thing-like experiential quality of the body in such cases is not the result of *objectifying* measures brought about by the doctor or the person himself.

The distinction between good and bad forms of objectification in medicine could be rephrased as the difference between cases of being perceived as a physical body without ceasing to be acknowledged as a lived and expressive body and the cases in which this acknowledgement is lacking. How is this acknowledgement brought about by the medical staff when examining the patient’s body? It is obviously not only a question of *talking* to the patient in the sense of using certain phrases like – “How do you feel?”, “Does this hurt?”, or “I am now inserting this instrument into your rectum, it is not dangerous but may feel a bit cold and strange.” – since all these words could be used in a purely instrumental way staying wholesale in the scientific investigative mode. To establish a relationship with the patient, the doctor needs time. It obviously helps if the examination has been preceded by a series of meetings during which trust has been established over months or years, and it also helps if the encounter itself is long enough to include a dialogic prelude and aftermath to the investigation.

To talk with the patient during a medical investigation is obviously a good idea, for various reasons, but the acknowledgement of the lived-body dimension could in some cases be brought about without any form of conversation. It could be accomplished by simply looking the patient in the eyes. The medical, objectifying gaze is humanized by the doctor, or nurse, by searching for and meeting the glance of the patient and seeing in his eyes that he is scared or confused or pain stricken or whatever. To perceive and acknowledge these feelings means to have and show empathy and such a second- instead of non-person point of view – that acknowledges the patient as a person in the medical meeting by way of his lived-expressive body – makes all the difference in the world (Halpern, 2001). If this empathic vigilance is lacking on the side of the medical personnel, the patient is in danger of being objectified in a bad manner.

## III. THE OBJECTIFYING GAZE

The medical-scientific gaze is not the only type of gaze that can objectify a person. Feminists have long thematized the male-sexist, objectifying gaze. In a classic paper, Martha Nussbaum (1995) identifies seven different ways in which women can be objectified by men (and in some cases men by women or same-sex objectification) in various situations. On her list are: being regarded as a tool, being denied autonomy, being denied agency, being regarded as interchangeable, being violated, being owned, and being denied subjectivity. As Nussbaum notes, these categories are neither conceptually nor empirically exclusive in the sense that they do not divide objectification into seven conceptual areas separated by neat lines. The areas are conceptually overlapping in various ways and, in addition to this, cases of objectification usually include more than one of these ways of being objectified. Some of Nussbaum’s categories clearly include more than an objectifying *gaze*, since the person being objectified is also *handled* – with hands or with words – in a way one treats an object, or perhaps a slave, rather than a subject. This is also, I think, how we should generally understand “gaze” when discussing objectification in everyday life as well as in medical practice.

The concept of the medical gaze was made famous by Michel Foucault in *The Birth of the Clinic* (1994). Foucault’s works are also important in the case of thematising a sexist gaze, but the feminist tradition has a much richer history (Nussbaum, 1995; Ahmed, 2006; Langton, 2009). A common source for both traditions is Jean-Paul Sartre’s phenomenology of the intersubjective gaze developed in *Being and Nothingness* (1992). According to Sartre, the gaze of the other person objectifies me, turns me into a body, and this is a painful experience since I am robbed of my subjective being. Sartre’s theory of the difference between conscious and material ways to exist – being-for-itself and being-in-itself – is complex, but his claim is, essentially, that we not only *become* ourselves by way of other people, but that we also *lose* ourselves by way of them, precisely by being turned into objects of their attention (Svenaeus, 2009). A less radical version of Sartre’s argument would be to claim that *some ways* of paying attention to the other person – by looking at her, talking to her, or handling her in some way – are objectifying in the ways we find on Nussbaum’s list. Some other ways of encountering the other person are not – for example, the ways assembled under the categories of empathy, sympathy, and love – and therefore we need a richer phenomenology than the one provided by Sartre to give social life its due. We could turn to other phenomenologists in search of fuller accounts of being towards and with the other, but this is not my mission in this paper (Szanto and Moran, 2015). Instead, I would like to come back to the question of whether there are bad *and* good ways of being objectified.

Bad ways of being objectified are characterized by attitudes making the objectified person feel that she is *merely* a physical body, as I put it in the introduction. It could be argued that a reference to the concerned person’s feelings is not enough to find out if objectification is going on or not, what is needed is an analysis of the institutional framework of medical – or sexist – processes of power. This is an argument found in works by scholars proceeding from a Hegelian-Marxist tradition blended with (post)structuralism (in the case of medical objectification, see, for instance Waitzkin and Britt, 1989). I would agree that the phenomenological tradition has suffered from, at least partial, blindness as it concerns the institutional framework of objectification. As concerns medical phenomenologists, a lot of energy has been invested to understand how medical science and technology developments objectify the symptoms experienced by patients, whereas the objectifying influence of health care bureaucracy and market models have not been brought to attention to a similar extent. Nevertheless, I think the cure for this blindness is not to abandon first-person experience as a source of truth concerning troubles of objectification, rather we should try to *extend* and *complement* the patient’s point of view by way of analyzing the context in which it occurs, in this case the clinical environment and the economic framework of medical technology development. This is what I will attempt to do below.

To return to Nussbaum’s list and her own discussion of it, it appears that the first item – treating a person as a tool – is the most problematic as far as morality is concerned. The ban on instrumentalizing human beings, treating them as mere means to an end, is the core of enlightenment ethics and famously conceptualized in Kant’s practical philosophy. From Nussbaum’s list, we could add: being regarded as interchangeable, being owned, and being denied autonomy or subjectivity as likewise interfering with Kant’s prohibition, although Nussbaum attempts to find legitimate cases of these ways of objectifying in certain contexts and practises (sexual life is her main theme in the paper).

By choosing “being regarded as a tool” as the worst way of being objectified, Nussbaum is not trying to say that being denied agency, and being violated, in the sense of having one’s mental and bodily boundaries broken, are not in most cases serious concerns for moral philosophy. She is trying to say that when asking ourselves if a case of sexual objectification is good or bad, much depends on context and consent. Being regarded as a tool is worse than, for instance, being violated – which sounds worse – since the latter, as well as being denied agency, could be a consented to and enjoyed ingredient of certain sexual practices, like BDSM. Being regarded as a tool – and possibly also being regarded as interchangeable, being owned, and being denied autonomy or subjectivity – is harder to make good by way of consent and context than being violated and denied agency, since it leaves no subject left to consent in the sexual act of being objectified. The subject becomes merely an object if she becomes a *mere* tool or *any* subject, in contrast to a subject that consents to and enjoys being objectified in other ways in the context of sexual life. Another way to phrase this is that the interchangeable tool-quality makes a person not only into an object but into a *commodity*, and this is also the logic that fuels sexist objectification of women in capitalist economies. According to this analysis, it would not be the asymmetrical power relation of the objectifying sexual acts that make them essentially bad but the *commodifying* quality of the objectification. The commodifying aspect of sexist objectification predates modern capitalism and it was (is) surely present also in socialist societies. Nevertheless, the commodifying qualities of sexist objectification fit very nicely into a capitalist framework, which fuels objectification of women despite the political development of women rights that has taken place in democratic societies.

## IV. BECOMING AN OBJECT THROUGH THE MEDICAL GAZE

Allow me now to come back to objectification in and through medical practice and technological development. The meetings between doctors, or other medical personnel, and patients in health care centers or hospitals are clearly *asymmetrical* relations, since the doctor is more knowledgeable and at home in comparison with the patient, who is suffering and vulnerable due to his condition (Pellegrino and Thomasma, 1981; Svenaeus, 2017). The patient in modern health care is a *visitor*, in contrast to the health care personnel who are at home in the clinic. This has not always been the case; in pre- and early-modern medicine it was the doctor who visited the patient in his home, and, as I will come back to, the future of medical practice may bring a similar shift of host and visitor (Topol, 2015).

For roughly the last 50 years, attempts have been made to make the clinical encounter more symmetrical by strengthening patients’ rights by way of laws and regulations, thereby making “medical paternalism” a species of the past in contrast to the ideal of “enhancing patient autonomy” (Jonsen, 1998). This means that the patient’s worries and wishes should be addressed in the encounter, that he has the right to know about his medical condition, and the right to refuse any treatment proposed by the doctor. Some neoliberal versions of patient autonomy come close to regarding the medical meeting as a business transaction, in which we are obligated to protect and strengthen customer’s rights (Harrisson, 2004). Such models could also emphasize the right to choose between doctors and health care centers to maximize customer satisfaction. The problem with such models is that they tend to strengthen the rights and wishes of some (rich and well educated) patients at the expense of other (poor and uneducated) ones. Resources for health-care services have always been limited in relation to the need for them, even in rich countries, and the contemporary development of increasingly more sophisticated, but also more expensive, diagnostic technologies, and medical therapies has far from solved this problem (Marmot, 2015).

Despite the attempts to enhance patient autonomy, medical encounters remain at heart asymmetrical in structure, as manifested in the objectification of patients taking place through physical examinations of their bodies, to a large extent made possible by way of diagnostic equipment (Reiser, 2009; Topol, 2015). Returning to Nussbaum’s list, we can say that the patient, if not being denied all autonomy, is at least being denied agency and having his bodily boundaries broken, in and through the physical examination. The medical gaze could be guilty of several of the objectifying transgressions mentioned in Nussbaum’s list, but as soon as we start thinking of the patient’s body, or parts thereof, as merely a tool – for instance, as transplantable or as fully interchangeable by making it a specimen of disease meant solely for research or education – we are entering the stage of commodification in addition to an asymmetrical relation of objectification within a certain context (Waldby and Mitchell, 2006; Leder, 2016). In contrast to this, the non-commodifying strategies of objectification in medical practice merely *display* the physiology and pathology of the biological organism to the medical staff, and this is done to be able to help the patient. The lived-bodily illness-experience of the patient, which is typically the reason for seeing the doctor in the first place, makes the patient vulnerable to commodification, especially if he is poor and could be exploited, but the good forms of medical objectification are necessary in order to help him and not necessarily alienating if contextualized in the proper way (Malmqvist and Zeiler, 2016). One should add that sexist or racist objectification could also pervert the medical meeting and in the medical context all forms of sexual or racist objectification – not only the commodifying ones – would be ethical violations.

In some cases, to make visible the physiological functions of the body by way of medical technological equipment could even *enhance* the feeling-at-home in and with one’s body rather than inducing a split. According to phenomenologists, illness is essentially a *not* feeling at home in and with one’s body (Svenaeus, 2019), a state in which one’s body feels *conspicuous, obtrusive, and obstinate*, to paraphrase Havi Carel (2008), a state in which the body *dys*- instead of disappears, to use a terminology invented by Drew Leder (1990). If I, by way of the physical examinations referred to above, get informed that what I suffer from is an inflammation of the stomach – a gastric ulcer, treatable by way of antibiotics – I might feel relief, and to be shown the pictures taken by way of endoscopy of my stomach from the inside will likely be interesting rather than frightening for me. If, in contrast to the first scenario, I get informed that what I suffer from is colon cancer, to be shown photographs of the tumor taken by way of the colonoscopy will likely make me feel even less at home with my body than I did before. The pains have now been objectified in a way that makes them even more frightful, my body increasingly becoming a territory that I do not control and which is being invaded by foreign malign powers.

While the details regarding the experience of being shown photographs of one’s intestines will vary with person and situation, the information that the disease is treatable or harmless will generally make it a less alienating experience. However, the mere instance of “having found something” by way of a medical examination will make the patient more vulnerable to experience illness even if he did not do so before the investigation. For example, being shown herniated disks of the spine by way of an MRI may lead to aggravated back pain despite the herniations not being responsible for the pain in question.

## V. TO LIVE BY WAY OF IMAGES AND MEASUREMENTS OF THE BODY

To be shown the insides of a human body is normally a terrifying experience, if not put into context and guided by the medical gaze. It is easier to obtain this frame of mind if the body which is opened up is not too close and present in flesh, a distancing maneuver which can be obtained by technological measures, as in the examples above, or, for instance, when we watch a medical documentary on the workings of the bowel system or a splatter movie. Only in the contexts of war, street violence, or traffic accidents do “ordinary people” – that is, persons who do not work in the health care sector – get to know bodies that expose their insides IRL. That bodies have an inside and not only an outside is something we are better informed about than any generation before us – because of medical science and technology development – but it is a fact that we do not generally *experience* by way of encountering dismembered and bleeding bodies in person, if we do not live in in a war zone, that is.

Citizens of rich countries are generally spared the everyday witnessing of ill and injured bodies that look, sound, and smell in distressing ways, including their own bodies and the bodies of their close relatives. Such business is taken care of by professionals in nursing homes or hospitals. However, new medical and information technologies make the insides of rich and privileged people’s bodies visible in other ways; ways that are not immediately frightening or disgusting, but nevertheless potentially disconcerting (Clarke et al., 2010). Medical companies offer diagnostic tests for biomarkers that reveal early-stage diseases or risks for developing diseases, and being informed about such test results – sometimes by being shown pictures, more often numbers – could lead to bodily alienation although there are no illness symptoms present yet (Hofmann and Svenaeus, 2018). Being told that one has a gene that predisposes her to develop breast cancer will make a person feel less at home with her body and provoke the question if she should opt for a preventive mastectomy – getting rid of parts of her body – or not. A male patient, being told that he has an enlarged prostate gland with possible malignity, will face a similar dilemma. Since such tests are increasingly targeted at genes or other biomarkers that predict risks of lesser magnitude than in the cases of the breast and prostate cancer, the question is how to balance the potential benefits of preventing disease with all the worries, hard choices, and other negative effects that may occur in the everyday life of the patient (Plomin, 2018).

Overuse of medical diagnostic procedures made possible by new technologies will lead patients – they become patients when they enter the diagnostic procedures regardless of being ill or not – to feel less at home in and with their own bodies in the sense that they focus on the body made present by the medical gaze rather than trust their own embodied experiences. This is often named *medicalization* since the objectification of the body as diseased, or at risk for disease, trumps the bodily feeling of wellness (Conrad, 2007). However, it is better to use the concept of *over diagnosis* in these cases, since medicine should arguably work on detecting and fixing everything that is health related – and the tests I have discussed so far are related to the (future) health of the patient – reserving the concept of medicalization for detecting and changing characteristics that go beyond health (Hauskeller, 2013; Parens, 2015). Regardless of terminology, the important distinction is between the cases when medicine should intervene – for instance, in detecting a tumor in my colon that could be surgically removed – and the cases when it should not – for instance when detecting a gene that increases risk for developing colon cancer by 5% but with few preventive measures available beyond general diet recommendations, which would prevent ill health in general.

A highly relevant development in the field of medical objectification are self-tracking options made use of by individuals subscribing to the so-called quantified-self movement (Topol, 2015). People are increasingly making use of wearable sensors that measure bodily parameters, such as heartbeat, temperature, blood sugar, movement, sleep pattern, diet, and so on to objectify the states of their bodies. The idea of providing numbers for bodily characteristics by way of technology and ordering them in various ways is certainly not new – think about height and weight measurements over time – but the detailed and massive collection of data which is attained by the “patients” themselves in contrast to health care workers and scientists is. Self-quantifying could arguably give rise to either enhanced bodily presence by working on your numbers and learning to control them or self-alienation when numbers replace lived bodily self-presence. The pressing question is if measurement and visualization of the bodily numbers increase or decrease the level of trust persons have in their own bodies. Do the numbers make the body more real or rather more foreign in self-quantifying? The risk seems to be that the natural foreignness of the lived body – I belong to my own body just as much as it belongs to me – gets replaced by a technological attitude towards my body that makes me yearn for a form of control I can never in reality attain (Slatman, 2014). The numbers also become exploitable when shared with other users or companies that hope to profit from the body surveillance movement. When this happens, the self-tracking measurements are not only objectifying the bodies of the users but also commodifying them.

## VI. MEDICAL HERMENEUTICS AND GOOD VERSUS BAD OBJECTIFICATIONS

Medical phenomenologists have developed the idea that medicine is essentially a *hermeneutical* practice (Gadamer, 1996; Svenaeus, 2000, 2017; Leder, 2016). To be a good doctor or a good nurse (or other good health care professional) one needs to be empathic – one needs to be able to feel and understand the fears, thoughts, and wishes of patients to help them in the best possible way. The first-person perspective of the patient must be acknowledged and understood from the point of view of the professional, making it into a second-person perspective, empathically and dialogically explored. To this second-person perspective a third- (or rather non-) person, medical-scientific perspective is added when exploring the biological functions of the potentially diseased body and the way the bodily parameters are related to the illness experience of the patient.

An additional perspective to be taken into account is the institutional setting of the health-care encounter, making it dependent on economic and political structures that shape and constrict the possibilities to help patients. This is not a perspective that doctors and other health care personnel as a rule actively adopt when trying to find out what is amiss with the patient – in the way they adopt a second-person and medical-scientific perspective – but it still shapes the encounters with patients in bad or good ways: in a bad way when it means lack of time and resources to meet with and help patients in need of medical assistance; in a good way when it means that the patient’s social and economic situation can in some way be taken into account in figuring out what needs to be changed to make him healthy. Since doctors and nurses are generally not in the position to change things like unemployment or lack of education, matters that are very clearly related to health in the long run, the institutional perspective is mainly a resource to be put to work by professionals in the area of public health and health politics (Marmot, 2015).

The first book that attempts to work out a modern philosophy of medicine based on the view that the healing relationship between physician (or other health care staff) and patient is the central feature of good medicine, in contrast to finding this core feature in the application of medical science and technology development, was *A Philosophical Basis of Medical Practice: Toward a Philosophy and Ethic of the Healing Professions* by Pellegrino and Thomasma (1981). Pellegrino and Thomasma articulate their philosophical paradigm of praxis in view of the development of what they call “medical economics,” which they think is threatening the ethical and professional standards of medical practice in the contemporary world (particularly in the USA) (1981, 266 ff.). Pellegrino’s and Thomasma’s books – *A Philosophical Basis of Medical Practice* (1981) is followed up by *The Virtues in Medical Practice* (1993) – may be considered as precursors to medical hermeneutics, since they trace the patterns of the medical meeting and makes it the hub of medical practice. The authors never use the term “hermeneutics” but they do use the term “phenomenology” and refer to Edmund Husserl and Maurice Merleau-Ponty in presenting the distinction between the lived and the physical body of the patient surveyed above (Pellegrino and Thomasma, 1981, 53, 74). According to Pellegrino and Thomasma medicine is: “*a relationship of mutual consent to effect individualized well-being by working in, with, and through the body*” (1981, 80). This is very much in line with the hermeneutic philosophy of medical practice that I have developed in *The Hermeneutics of Medicine and the Phenomenology of Health: Steps towards a Philosophy of Medical Practice* and I also make use of the authors’ definition in the book (Svenaeus, 2000, 52).

The kind of hermeneutics that is essential to medicine as an empathic and dialogic way of interpreting the stories told by patients is a *phenomenological* hermeneutics (Svenaeus, 2000). Such a hermeneutics was first developed by Martin Heidegger in his main work, *Being and Time* (1996), and taken further by Hans-Georg Gadamer in *Truth and Method* (1994) and by Paul Ricoeur in *Oneself as Another* (1992), but none of them include medicine in the phenomenological-hermeneutic endeavor. According to such a hermeneutic view, medical practice is an understanding activity which is identical neither with explanation in the sciences nor with interpretation in the humanities. Medical knowledge includes applied biology – scientific explanations of what happens in the diseased body – but is not limited to this scientific approach. The hub of medical hermeneutics is the dialogue between health care professional and patient that represents a particular form of understanding in and by which all forms of particular scientific investigations are guided (or, at least, should be guided) (Gadamer, 1996). The medical encounter can be viewed as a coming-together of the two different attitudes and worlds of health care professional and patient – in the language of Gadamer, of their different horizons of understanding – aimed at establishing a mutual understanding which can benefit the health of the ill party. Doctors (as well as representatives of other health care professions) are thus not first and foremost scientists who apply biological knowledge but, rather, interpreters – hermeneuts of health and illness. Biomedical explanations and therapies can only be applied *within* the dialogical meeting, guided by the clinical understanding attained in service of the patient and his health.

What is particularly obvious in the medical meeting, as I have continuously pointed out, is the *asymmetrical* relation between the parties. The patient is ill and seeks help, whereas the doctor is at home − in control by virtue of her knowledge and clinical experience of disease and illness. As developed above, this asymmetry necessitates empathy on the part of the doctor (and other health-care personnel). She must try to understand the patient, not exclusively from her own point of view, but through trying to put herself in the patient’s situation by listening to his story. Consequently, that the doctor attempts to reach a new, productive understanding of the patient’s illness in no way implies that she should avoid empathy. It is only through empathy that the doctor can reach an independent understanding that is truly productive, in the sense of shared *and* novel, in offering new perspectives on the patient’s health problems (Svenaeus, 2017, Chapter 4).

A major problem for medical hermeneutics in the contemporary culture of self-measurement and direct-to-consumer genetic testing is that an increasing number of medical objectifications are taking place *outside* the clinic and are being interpreted by the patients themselves. This could be viewed as a strengthening of patient autonomy (see above) making the patient more independent of doctors and other health care professionals, but it also gives rise to worries, since the patient is not always able to understand and evaluate the objectifications of his body being made and presented to him. Sharing data via health-apps and DNA-tests made available by med-tech companies may lead to commodification of persons’ bodies and it may also lead to bad medical objectification in the sense that persons feel less at home with their bodies than before the measurements and tests took place. To interpret medical data a thorough hermeneutics is called for, not only informed by the experiences and life story of the patient, but also by in-depth biomedical knowledge and statistics in relation to risks. Such a hermeneutics could be provided by strengthening the bonds between patients and physicians in primary health care, a setting in which the need for and results of medical tests could be judged and interpreted in a professional manner, making the patients less vulnerable to bad medical objectification and exploitation.

## VII. CONCLUSION

I have identified at least four ways in which medical-technological objectification of a person’s body could lead to experiencing increased foreignness and distress. Medical objectification may lead to bodily alienation by revealing asymptomatic signs or markers for a disease present in the body (made present by way of a medical image or a laboratory result); it can reveal risk factors for developing diseases in the future (genetic or other biomarker testing); it may affect and increase an already present illness experience (by way of images or numbers); and it may influence and change our experiences of health. Technologies that influence and change experiences of being healthy in detrimental ways are found in the cases of self-tracking – wearable sensors and digital measurements – making deviations from ideal patterns more visible and possibly adopting goals that fail to come true by the way of the numbers.

In all these cases, doctors and other medical professionals could help persons to make sense of different forms of objectifications of their bodies and thereby make it less likely that images and numbers are interpreted and experienced in alienating ways. Medical professionalism in the contemporary scenario is not the problem but rather the cure against unhealthy technological developments, provided the professionals adopt a hermeneutical view of their mission. Considering the future not only of apps for health-related self-tracking, but also of genetic and other biomarker tests for various disease risks available from direct-to-consumer companies, it is of vital importance not to leave patients alone with the increasing number of medical objectifications they will potentially have to take into account in their everyday lives. Not only is widespread commodification of patients’ bodies lurking around the corner, but also an increasing confusion among patients about how to interpret and live with a large number of medical risks and yet stay at home in and with the body. This is the contemporary scenario in which it has become urgent to understand how different forms of medical objectification work and are to be handled so as to enhance rather than alienate patients’ lives. Phenomenology, as I have tried to show in this paper, could be of assistance in such an endeavor by distinguishing different forms of objectification – medical in contrast to sexual or racial objectification – and good versus bad ways of putting them to work in the clinical encounter.
